# Supplementary material for: Treatment with bulevirtide in HIV-infected patients with chronic hepatitis D: ANRS HD EP01 BuleDelta and compassionate cohort
Source: JHEP Rep. 2024 Mar 26;6(8):101057. doi: 10.1016/j.jhepr.2024.101057 (PMC11264178; doi:10.1016/j.jhepr.2024.101057)
Supplement: Multimedia component 1 [file mmc1.pdf]

Treatment with bulevirtide in HIV-infected patients with chronic hepatitis D: ANRS HD EP01 BuleDelta and compassionate cohort

Victor de Lédinghen, Claire Fougerou-Leurent, Estelle Le Pabic, Stanislas Pol, Dulce Alfaiate, Karine Lacombe, Marie-Noëlle Hilleret, Caroline Lascoux-Combe, Anne Minello, Eric Billaud, Isabelle Rosa, Anne Gervais, Vlad Ratziu, Nathalie Ganne, Georges-Philippe Pageaux, Vincent Leroy, Véronique Loustaud-Ratti, Philippe Mathurin, Julie Chas, Caroline Jezequel, Sophie Métivier, Jérôme Dumortier, Jean-Pierre Arpurt, Tarik Asselah, Bruno Roche, Antonia Le Gruyer, Marc-Antoine Valantin, Caroline Scholtès, Emmanuel Gordien, Christelle Tual, Amel Kortebi, Fatoumata Coulibaly, Eric Rosenthal, Miroslava Subic-Levrero, Dominique Roulot, Fabien Zoulim, and the ANRS HD EP01 BuleDelta study group

Table of contents

Fig. S1……………………………………………………………………………2

Table S1…………………………………………………………………………5

Table S2…………………………………………………………………………6

Table S3…………………………………………………………………………7

Supplementary references………………………………………………………..8

Fig. S1. Virological and/or biochemical response (intent to treat analysis)

A Virological response (≥ 2 log_{10} HDV RNA decline from baseline) at week 12, 24 and 48

B Undetectable HDV RNA at week 12, 24 and 48

C Biochemical response (normal ALT level) at week 12, 24 and 48

D Combined response (normal ALT level and ≥ 2 log_{10} HDV RNA decline from baseline or undetectable HDV RNA) at week 12, 24 and 48

BLV. Bulevirtide

PEG-IFN. Pegylated interferonα

Supplementary file 1A

Virological response (ITT)

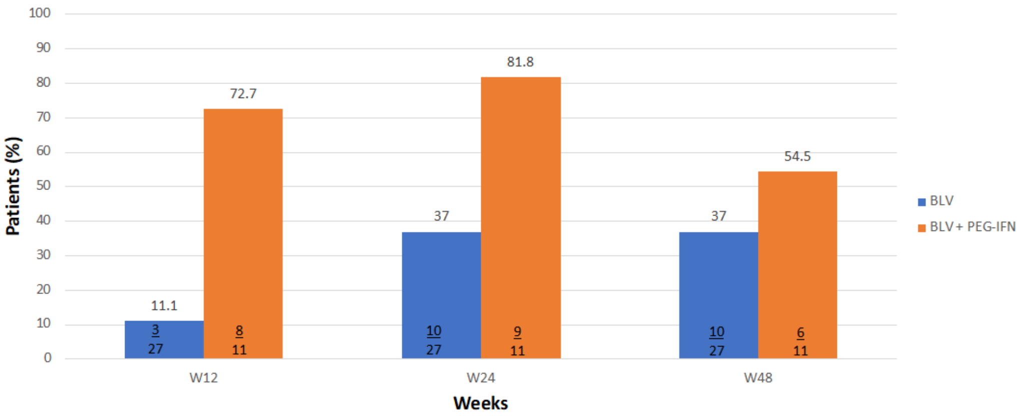

Supplementary file 1B

Undetectable HDV-RNA (ITT)

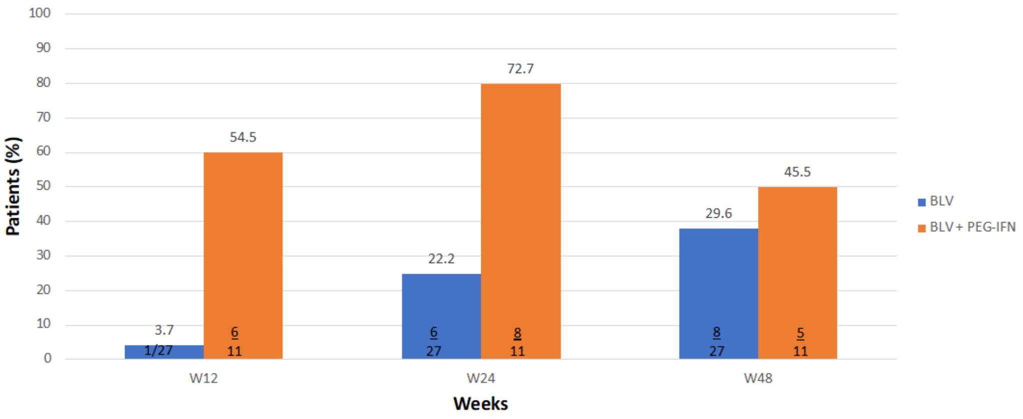

Supplementary file 1C

Biochemical response (ITT)

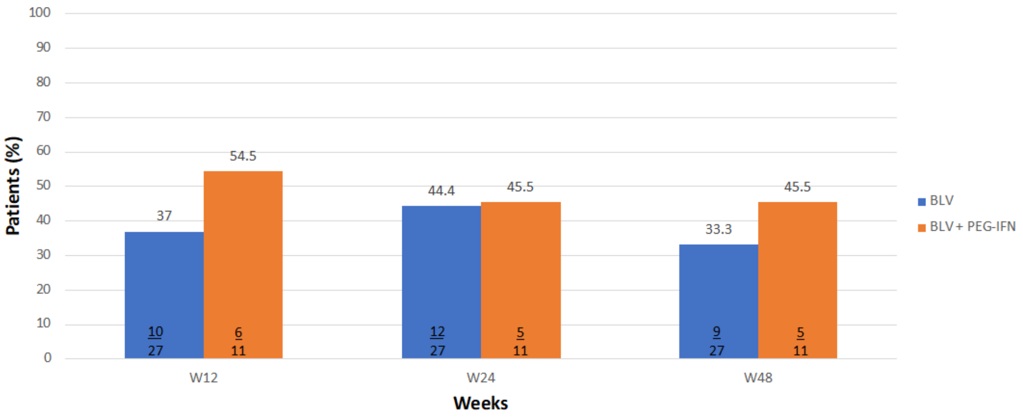

Supplementary file 1D

Combined response (ITT)

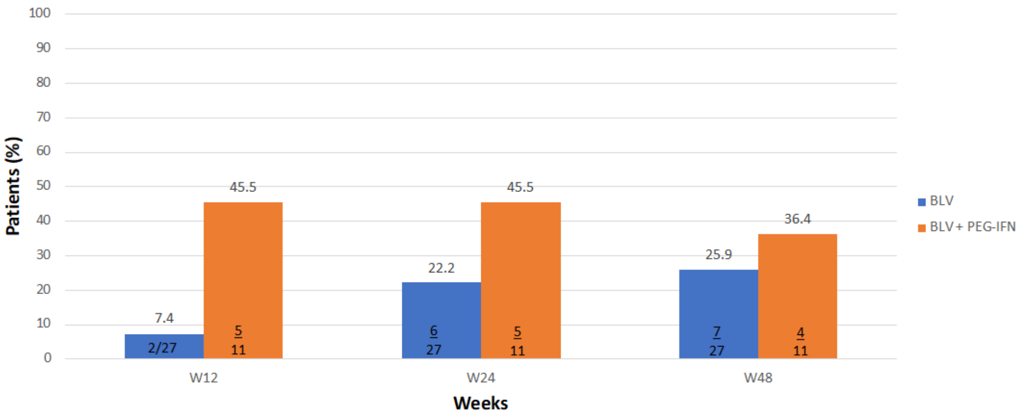

Table S1. Characteristics of Patients discontinuing antiviral therapy.

ID	Gender	Age (years)	HDV treatment	LSM (kPa)	Cirrhosis	HDV RNA at D0 (log_{10} IU/ml)	ALT level at D0 (IU/L)	Treatment duration (weeks)	HDV RNA at the end of treatment (log_{10} IU/ml)	ALT level at the end of treatment (IU/L)	Cause of discontinuation	HDV-RNA at FU12 (log_{10} IU/ml)	ALT at FU12 (IU/L)
1	Female	56	BLV	NA	Yes	7.27	57	35.86	6.44	20.5	Virological failure	6.96	78.9
2	Male	54	BLV + pegIFN α	75	Yes	6.22	100	3.86	6.33	65	Variceal bleeding	NA	NA
3	Male	52	BLV + pegIFN α	NA	No	4.71	63	16.86	4.26	46	NA	3.68	68
4	Female	55	BLV	20.9	Yes	5.22	62	25.86	3.51	33	Patient wish	NA	NA
5	Male	55	BLV	22	Yes	6.57	81	25.43	5.24	55	Lost to FU	NA	NA
6	Male	35	BLV	5.7	No	5.70	145	37.43	6.06	66	No compliance	5.69	95
7	Male	51	BLV + pegIFN α	70.1	Yes	4.67	36	24.00	0	21	Adverse events	4.36	70
8	Male	42	BLV + pegIFN α	22	Yes	3.99	75	11.71	0	42	Prison	0	33

FU: follow-up
LSM: liver stiffness measurement

Table S2. Characteristics of 11 patients with detectable HIV RNA at initiation of treatment or W48.

ID	Gender	Age (years)	HDV treatment	HIV RNA at D0 (cp/ml)	HIV RNA level detection (cp/ml)	CD4 at D0 (/mm^3)	HIV RNA at W48 (cp/ml)	CD4 at W48 (/mm^3)	HIV treatment
1	Male	55	BLV	0	20	480	30	524	TAF/FTC + INSTI + NNRTI
2	Male	30	BLV	65	20	656	0	735	TAF/FTC + INSTI
3	Male	59	BLV	33	20	128	NA	NA	TDF/FTC + INSTI
4	Male	39	BLV + pegIFNα	30	20	843	70	1033	TAF/FTC + NNRTI
5	Male	52	BLV + pegIFNα	21	20	710	NA	NA	TDF/FTC + protease inhibitor
6	Female	56	BLV	30	20	NA	NA	NA	TAF/FTC + INSTI
7	Male	55	BLV	50	20	375	0	293	TAF/FTC + INSTI
8	Female	48	BLV	123	20	588	238	544	TDF/FTC + INSTI + CCR5 antagonist
9	Female	41	BLV	20	20	317	NA	NA	TDF/FTC + protease inhibitor
10	Male	45	BLV	19000	20	179	NA	NA	3TC + INSTI
11	Male	51	BLV + pegIFNα	31	20	500	54	470	TAF/FTC + INSTI

INSTI: Integrase strand transfer inhibitor
NNRTI : Non-nucleoside reverse ratzscriptase inhibitor
BLV: Bulevirtide; BLV + pegIFNα: Bulevirtide + pegylated interferon alfa;
TAF: Tenofovir alafenamide; FTC: Emtricitabine; TDF: Tenofovir Disoproxil Fumarate; CCR5: Chemokine receptor 5; 3TC: Lamivudine; NA: Not available

Table S3. Characteristics of the 11 patients with no response or partial response at week 48.

ID	Gender	Age (years)	HDV treatment	LSM (kPa)	Cirrhosis	HDV RNA at D0 (log10 IU/ml)	HDV RNA at W48 (log10 IU/ml)	HDV RNA at W60 (log10 IU/ml)	HDV RNA at W72 (log10 IU/ml)	HDV RNA at EOT (log10 IU/ml)
		Non responder patients								
1	Male	55	BLV	38.5	Yes	5.74	5.42	6.07	NA	
2	Male	44	BLV	NA	Yes	5.36	5.78	NA	NA	
3	Male	40	BLV	NA	Yes	6.78	6.44	5.52	NA	
4	Female	35	BLV	7.9	No	7.19	7.71	5.16	0	
5	Male	33	BLV	NA	No	6.35	5.71	NA	NA	6.52 (W87)
6	Female	48	BLV	9.9	Yes	5.15	4.48	5.29	NA	
		Partial responder patients								
7	Male	39	BLV+ pegIFNα	4.2	No	6.85	5.60	NA	4.60	
8	Male	58	BLV	6.1	Yes	6.74	5.19	5.75	6.05	
9	Male	55	BLV	48.8	Yes	7.90	6.5	5.95	NA	
10	Female	42	BLV+ pegIFNα	9.5	No	6.32	5.12	NA	NA	5.30 (W59)
11	Male	46	BLV	15	Yes	5.17	3.89	4.32	4.10	

*EOT = End Of Treatment

LSM: liver stiffness measurement

Supplementary references

1. Asselah T, Rizzetto M. Hepatitis D Virus Infection. N Engl J Med 2023;389:58-70.
2. Piroth L, Pol S, Lacombe K, et al. Management and treatment of chronic hepatitis B virus infection in HIV positive and negative patients: the EPIB 2008 study. J Hepatol 2010;53:1006-1012.
3. Stockdale AJ, Kreuels B, Henrion MYR, et al. The global prevalence of hepatitis D virus infection: Systematic review and meta-analysis. J Hepatol 2020;73:523-532.
4. Alfaiate D, Clement S, Gomes D, et al. Chronic hepatitis D and hepatocellular carcinoma: A systematic review and meta-analysis of observational studies. J Hepatol 2020;73:533-539.
5. **Wedemeyer H, Yurdaydin C**, Hardtke S, et al. Peginterferon alfa-2a plus tenofovir disoproxil fumarate for hepatitis D (HIDIT-II): a randomised, placebo controlled, phase 2 trial. Lancet Infect Dis 2019;19:275-286.
6. Martin P, Jacobson IM. New Therapies and Management Options for Hepatitis D. Am J Gastroenterol 2023;118:1105-1107.
7. Lampertico P, Roulot D, Wedemeyer H. Bulevirtide with or without pegIFNalpha for patients with compensated chronic hepatitis delta: From clinical trials to real-world studies. J Hepatol 2022;77:1422-1430.
8. Wedemeyer H, Schoneweis K, Bogomolov P, et al. Safety and efficacy of bulevirtide in combination with tenofovir disoproxil fumarate in patients with hepatitis B virus and hepatitis D virus coinfection (MYR202): a multicentre, randomised, parallel-group, open-label, phase 2 trial. Lancet Infect Dis 2023;23:117-129.
9. Wedemeyer H, Aleman S, Brunetto MR, et al. A Phase 3, Randomized Trial of Bulevirtide in Chronic Hepatitis D. N Engl J Med 2023;389:22-32.
10. Dietz-Fricke C, Tacke F, Zollner C, et al. Treating hepatitis D with bulevirtide - Real-world experience from 114 patients. JHEP Rep 2023;5:100686.
11. Degasperi E, Anolli MP, Uceda Renteria SC, et al. Bulevirtide monotherapy for 48 weeks in patients with HDV-related compensated cirrhosis and clinically significant portal hypertension. J Hepatol 2022;77:1525-1531.
12. European Association for the Study of the Liver. Electronic address eee, European Association for the Study of the L. EASL Clinical Practice Guidelines on hepatitis delta virus. J Hepatol 2023;79:433-460.
13. Visco Comandini U, De Santis E, De Maria F, et al. "Real world" efficacy of bulevirtide in HBV/HDV-related cirrhosis including people living with HIV: Results from the compassionate use programme at INMI Spallanzani in Rome, Italy. HIV Med 2023; Oct;24(10):1075-1082.
14. **Roulot D, Brichler S**, Layese R, et al. Origin, HDV genotype and persistent viremia determine outcome and treatment response in patients with chronic hepatitis delta. J Hepatol 2020;73:1046-1062.
