# Supplementary material for: Treatment with bulevirtide in HIV-infected patients with chronic hepatitis D: ANRS HD EP01 BuleDelta and compassionate cohort
Source: JHEP Rep. 2024 Mar 26;6(8):101057. doi: 10.1016/j.jhepr.2024.101057 (PMC11264178; doi:10.1016/j.jhepr.2024.101057)
Supplement: Multimedia component 4 [file mmc4.pdf]

Research article

JHEP Reports

Treatment with bulevirtide in HIV-infected patients with chronic hepatitis D: ANRS HD EP01 BuleDelta and compassionate cohort

Authors

Victor de Lédinghen, Claire Fougerou-Leurent, Estelle Le Pabic, …, Miroslava Subic-Levrero, Dominique Roulot, Fabien Zoulim

Correspondence

professeur.deledinghen@gmail.com (V. de Lédinghen).

Graphical abstract

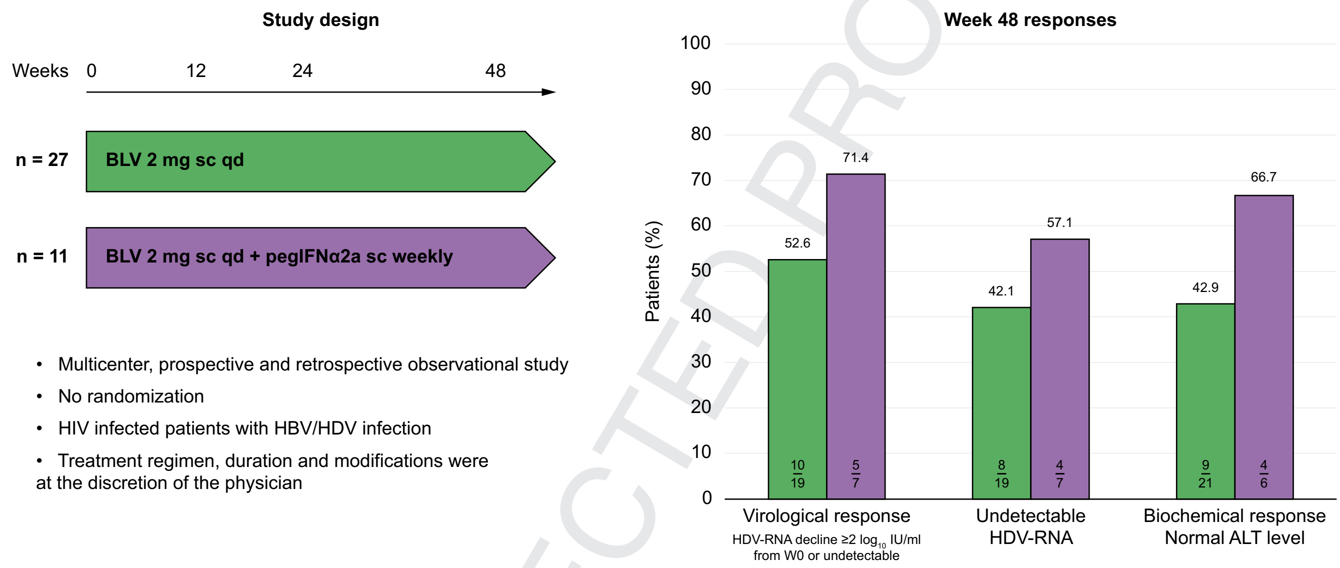

Highlights:

- Bulevirtide is a new treatment for HDV infection, but its efficacy and safety in HIV-coinfected patients are unknown.
- Treatment of hepatitis delta with bulevirtide in HIV-infected patients is safe, with no specific drug–drug interactions.
- A virological response was observed in >50% of patients.
- In clinical practice, bulevirtide can be used in HIV-coinfected patients, though the ideal duration of treatment remains unknown.

Impact and implications

Bulevirtide is the only EMA-approved drug for HDV treatment, and we showed that it can be used in adults living with HIV, with an overall good tolerability. Bulevirtide induces a virological response in more than 50% of patients, suggesting that bulevirtide should be considered as a first-line therapy in this specific population. Bulevirtide in combination with pegIFNα could be used in patients without pegIFNα contraindication. No specific drug–drug interaction is reported.

UNCORRECTED PROOF

https://doi.org/10.1016/j.jhepr.2024.101057

© 2024 The Author(s). Published by Elsevier B.V. on behalf of European Association for the Study of the Liver (EASL). This is an open access article under the CC BY license (http://creativecommons.org/licenses/by/4.0/).

Treatment with bulevirtide in HIV-infected patients with chronic hepatitis D: ANRS HD EP01 BuleDelta and compassionate cohort

Victor de Lédinghen[1,*], Claire Fougerou-Leurent[2], Estelle Le Pabic[2], Stanislas Pol[3], Dulce Alfaiate[4], Karine Lacombe[5], Marie-Noëlle Hilleret[6], Caroline Lascoux-Combe[7], Anne Minello[8], Eric Billaud[9], Isabelle Rosa[10], Anne Gervais[11], Vlad Ratziu[12], Nathalie Ganne[13], Georges-Philippe Pageaux[14], Vincent Leroy[15], Véronique Loustaud-Ratti[16], Philippe Mathurin[17], Julie Chas[18], Caroline Jezequel[19], Sophie Métivier[20], Jérôme Dumortier[21], Jean-Pierre Arpurt[22], Tarik Asselah[23], Bruno Roche[24], Antonia Le Gruyer[25], Marc-Antoine Valantin[26], Caroline Scholtès[27], Emmanuel Gordien[28], Christelle Tual[2], Amel Kortebi[2], Fatoumata Coulibaly[29], Eric Rosenthal[29], Miroslava Subic-Levrero[30], Dominique Roulot[13], Fabien Zoulim[30], the ANRS HD EP01 BuleDelta study group

JHEP Reports **2024**. vol. 6 | 1–7

Check for updates

Background & Aims: In France, bulevirtide (BLV) became available in September 2019 through an early access program to treat patients with HDV. The aim of this analysis was to evaluate the efficacy and safety of BLV in patients with HIV and HDV coinfection.

Methods: Patients received BLV 2 mg ± pegylated interferon-α (pegIFNα) according to the physician's decision. The primary endpoint (per-protocol analysis) was the virological response rate at Week 48, defined as the proportion of patients with undetectable serum HDV RNA or a HDV RNA decline >2 $\log_{10}$ IU/ml from baseline.

Results: The characteristics of the 38 patients were as follows: 28 male, mean age 47.7 years, and mean baseline HDV RNA viral load 5.7 ± 1.2 $\log_{10}$ IU/ml. Median HIV viral load and mean CD4 count were 32 (30–65) copies/ml and 566 ± 307/mm^3, respectively. Eight patients stopped treatment before Week 48. At Week 48, 10 of 19 patients (52.6%) in the 2 mg BLV group and five of seven patients (71.4%) in the 2 mg BLV + pegIFNα group had reached virological response (no HDV RNA available in four patients). At Week 48, seven of 19 patients in the 2 mg BLV group and three of six patients in the 2 mg BLV + pegIFNα group had a combined response (virological response and normal alanine aminotransferase level).

Conclusions: Adults living with HIV coinfected with HDV can be treated by BLV with a virological response in more than 50% of patients. The combination of BLV and pegIFNα showed a strong virological response.

© 2024 The Author(s). Published by Elsevier B.V. on behalf of European Association for the Study of the Liver (EASL). This is an open access article under the CC BY license (http://creativecommons.org/licenses/by/4.0/).

Introduction

HDV is a defective, hepatotropic pathogenic agent that requires the HBsAg provided by HBV.[1] Approximately 5% of individuals infected with HBV are coinfected with HDV, but this rate reaches 12% in HIV-coinfected patients.[2,3] Chronic HBV/HDV coinfection is associated with an unfavorable outcome, with many patients developing liver cirrhosis, liver failure, and eventually hepatocellular carcinoma within 5–10 years.[4]

Pegylated interferon-α (pegIFNα) has been used for treating patients with HDV for the last 30 years, with only limited sustained responses.[5] A 48-week course of weekly s.c. injections of pegIFNα suppresses HDV replication in approximately 20–30% of patients 24 weeks off therapy, with significant side effects. Continuous administration of pegIFNα for more than 48 weeks may lead to HBsAg loss in approximately 10% of these patients during long-term follow up.[6]

In July 2020, the entry inhibitor bulevirtide (BLV) received conditional marketing authorization in the European Union. BLV is a s.c. delivered lipopeptide that mimics the sodium taurocholate cotransporting polypeptide receptor binding domain of the L-HBsAg, inhibiting the HBV/HDV entry into the hepatocytes.[7]

In the phase II MYR202 study, treatment with BLV, at different doses during 24 weeks, was evaluated in 90 patients with chronic HBV/HDV coinfection.[8] The primary endpoint was undetectable HDV RNA or a 2 $\log_{10}$ IU/ml or greater decline in HDV RNA at Week 24. At Week 24, 15 (54%; 95% CI 34–73) of 28 patients achieved undetectable HDV RNA or a 2 $\log_{10}$ IU/ml or greater decline in HDV RNA with 2 mg BLV. By Week 48 (24 weeks after BLV cessation), HDV RNA concentrations had rebounded, with median changes from Week 24 to Week 48 of 1.923 $\log_{10}$ IU/ml (IQR 0.566–2.485 $\log_{10}$ IU/ml) with 2 mg BLV. In the phase III MYR301 study, only 12% of patients had un-

* Corresponding author. Address: Service d'Hepatologie, Hôpital Haut-Lévêque, 33604 Pessac Cedex, France. Tel.: +33-557-656-439; Fax: +33-557-656-445.
E-mail address: professeur.deledinghen@gmail.com (V. de Lédinghen).
https://doi.org/10.1016/j.jhepr.2024.101057

detectable HDV RNA after 48 weeks of BLV 2 mg. However, 71% of patients had a virological response defined by undetectable HDV RNA or a ≥2 log_{10} IU/ml decrease in HDV RNA level.[9] Many authors have published real-world experiences with similar results.[10,11] However, in these studies, the adjunction of pegIFNα to BLV was not evaluated. Lastly, the optimal dose and duration of BLV have not yet been defined, as stated in the recent EASL clinical practice guidelines.[12]

Only one study has evaluated the efficacy and tolerance of BLV in five patients with HIV.[13] In this short-term study with a small number of patients, at Month 6, a combined response (virological and biochemical) was achieved in 60% of patients.

In France, BLV became available in September 2019 through an early access program. The aim of this analysis was to evaluate the efficacy and safety of BLV in real life in consecutive HIV/HBV-coinfected patients with HDV infection.

Patients and methods

Patients

From September 2019, all consecutive patients enrolled in the French early access program were included in a follow-up cohort (compassionate cohort). From February 2020 to March 2022, the ANRS HD EP01 BuleDelta cohort (NCT04166266) included patients with hepatitis D treated with BLV. The inclusion criteria in the early access program were as follows: age >18 years, compensated cirrhosis or chronic hepatitis with at least moderate fibrosis (F2), and alanine aminotransferase (ALT) elevation. All patients treated with BLV could be included in the BuleDelta cohort. Information about the study was provided to all patients, and those included in the ANRS HD EP01 BuleDelta cohort gave written consent. In the present study, patients co-infected with HIV were selected from both cohorts.

Treatment

Patients received BLV 2 mg s.c. q.d. Treatment duration, its association with pegIFNα, and the dosage of pegIFNα, were at the physician's discretion. Most patients received 180 μg/week. Moreover, patients could be treated with or without nucleos(t)ide analog (NUC), at the physician's discretion.

Routine follow-up

Patients underwent routine laboratory tests, anti-HDV antibodies, HDV RNA, liver biopsy, or liver stiffness measurement (FibroScan®, Echosens, Paris, France). Cirrhosis was defined as a past history of ascites or variceal bleeding, a liver biopsy with a F4 stage (METAVIR score), liver stiffness measurement (FibroScan®) >11 kPa, or FIB-4 >3.25. Several HDV RNA assays were used across the clinical centers. Their lower limit of detection ranged from 120 to 1,000 copies/ml or from 100 to 1,000 IU/ml. Most analyses were performed using the EurobioPlex assay (Eurobio Scientific, Les Ullis, France).

The HIV RNA level of detection ranged between 20 and 40 copies/ml, depending on the clinical centers.

Recommended follow-up was every 3 months. However, because they were observational studies, the follow-up was left to the discretion of each physician. Follow-up assessments included blood sampling, vital signs, physical examination, and collection of adverse events (AEs). Grade 3 AEs and serious AEs (SAEs) were reported and documented in the BuleDelta cohort. They were evaluated by the investigators for seriousness, relatedness, and severity, according to the Common Terminology Criteria for Adverse Events version 4.03. All AEs and SAEs were reported in the compassionate cohort. All participants with AEs were followed up by the responsible investigator to report the outcome until complete resolution or stabilization.

Outcomes

The primary endpoint was the virological response rate at Week 48, defined as the proportion of patients with undetectable serum HDV RNA or an HDV RNA decline >2 log_{10} IU/ml from baseline. Secondary endpoints included change from baseline in HDV RNA levels at Week 48, undetectable HDV RNA at Week 48, combined response (defined as undetectable HDV RNA or an HDV RNA decline >2 log_{10} IU/ml from baseline and normal ALT) at Week 48, and changes from baseline in ALT at Week 48.

When treatment was continued after Week 48, virological and biochemical responses were evaluated at Weeks 72 and 96.

Statistical analysis

The evaluation of primary efficacy was based on per-protocol and intent-to-treat analyses. The per-protocol analysis was conducted on patients receiving treatment at the time of evaluation. The intent-to-treat analysis included all patients, with the non-evaluable patients considered as non-responders. Safety was assessed for all patients who received at least one dose of BLV.

Qualitative variables were reported as number and percentages, and quantitative variables were reported as mean ± SD or median (IQR). Logistic regression was used to identify factors associated with virological response at Week 48, and results were expressed as odds ratio (OR) with their 95% CIs. A test was considered statistically significant if the adjusted p value was less than 0·05. Statistics and graphs were generated using SAS 9.4 software (SAS Institute, Cary, NC, USA).

Results

Characteristics of patients

A total of 38 patients were included in this analysis (27 from the ANRS HD EP01 BuleDelta cohort and 11 from the compassionate cohort). The main characteristics of patients are detailed in Table 1 and are as follows: 28 male, mean age 47.7 ± 8.6 years, mean BMI 26.1 ± 6.3 kg/m^2. Mean baseline HDV RNA viral load was 5.7 ± 1.2 log_{10} IU/ml. Most patients (59.5%) had received pegIFNα in the past. Twenty-six (68.4%) patients had cirrhosis, including one patient with a past history of ascites. Median HIV viral load and mean CD4 count were 32 (30–65) copies/ml and 566 ± 307/mm^3, respectively.

A total of 27 patients were treated with BLV 2 mg, and 11 patients were treated with 2 mg BLV + pegIFNα. In addition, 30 patients completed 48 weeks of treatment: 23 (85.2%) in the 2 mg BLV group and seven (63.6%) in the 2 mg BLV + pegIFNα group. Viral load was not available at Week 48 in four patients. Eight patients stopped treatment before Week 48. Reasons for early termination before Week 48 are listed in Table 2. Median follow up after treatment initiation was 83 weeks (extremes,

Table 1. Characteristics of the 38 patients at inclusion.

	All patients (N = 38)	BLV monotherapy (n = 27)	BLV + pegIFNα (N = 11)	*p* value
Age (years), mean ± SD	47.7 ± 8.6	47.7 ± 9.8	47.8 ± 5.1	0.9627
Male, n (%)	28 (73.7)	21 (77.8)	7 (63.6)	0.4318
BMI (kg/m^2), mean ± SD	26.1 ± 6.3	24.6 ± 3.9	29.2 ± 9.1	0.0589
CD4 count ($cells/mm^3$), mean ± SD	566.2 ± 306.6	583.4 ± 331.0	524.2 ± 249.0	0.6340
HIV RNA (copies/ml), median (IQR)	32 (30–65)	50 (30–123)	30 (21–31)	0.2086
Patients with quantifiable HIV RNA, n (%)	10 (26.3)	7 (25.9)	3 (30)	1.0000
Time since HDV diagnosis (years), mean ± SD	11.3 ± 9.0 *11	10.8 ± 9.0 *6	13.3 ± 9.3 *5	0.5533
Past history of hepatocellular carcinoma, n (%)	1 (2.6)	1 (3.7)	0 (0)	1.0000
Liver stiffness measurement (kPa), median (IQR)	10.1 (7.9–15) *9	9.3 (6.3–15) *8	13.2 (9.5–22) *1	0.1832
FIB-4, mean ± SD	3.3 ± 2.7 *1	3.5 ± 3.1	2.6 ± 1.4 *1	0.3778
Cirrhosis, n (%)	26 (68.4)	18 (66.7)	8 (72.7)	1.0000
Previous use of pegIFNα, n (%)	22 (59.5) *1	15 (55.6)	7 (70) *1	0.4806
Platelets (G/L), mean ± SD	152.9 ± 54.0	152.8 ± 58.3	153.3 ± 44.1	0.9815
AST (IU/L), mean ± SD	80.0 ± 40.1 *1	86.1 ± 41.6	63.7 ± 31.7 *1	0.1329
ALT (IU/L), mean ± SD	101.7 ± 65.6	113.4 ± 70.8	73.0 ± 40.2	0.0852
Normal ALT, n (%)	5 (13.2)	2 (7.4)	3 (27.3)	0.1341
GGT (IU/L), mean ± SD	100.7 ± 97.0 *5	99.5 ± 101.0 *3	103.9 ± 91.0 *2	0.9091
Total bilirubin (μmol/L), mean ± SD	11.7 ± 7.4 *2	11.8 ± 7.6 *1	11.3 ± 7.1 *1	0.8499
Albumin (g/L), mean ± SD	37.8 ± 3.6 *9	37.7 ± 3.5 *7	38.1 ± 3.8 *2	0.7653
Negative HBeAg, n (%)	28 (87.5)	17 (81.0)	11 (100)	0.2720
Undetectable HBV DNA, n (%)	23 (62.2) *1	16 (59.3)	7 (70.0) *1	0.8007
NUC treatment, n (%)	37 (97.4)	26 (96.3)	11 (100)	1.0000
qHBsAg (IU/ml), mean ± SD	6,117.8 ± 10,208 *9	5,935.1 ± 10,643 *8	6,465.0 ± 9,869 *1	0.8971
HDV RNA (log_{10} IU/ml), mean ± SD	5.7 ± 1.2	5.7 ± 1.3	5.7 ± 1.0	0.9311
HDV genotype, n (%)	*19	*11	*8	0.0103
1	14 (73.7)	14 (87.5)	0 (0)	
5	4 (21.1)	2 (12.5)	2 (66.7)	
7	1 (5.3)	0 (0)	1 (33.3)	
HIV treatment, n (%)				
3TC	3 (8.1)	3 (11.5)	0 (0)	
TAF/FTC	22 (59.5)	15 (57.7)	7 (63.6)	
TDF/FTC	12 (32.4)	8 (30.8)	4 (36.4)	
INSTI	23 (62.1)	16 (61.5)	7 (63.6)	
NNRTI	12 (32.4)	9 (34.6)	3 (27.3)	

Qualitative variables are reported as n (%), and quantitative variables are reported as mean ± SD or median (IQR). Student *t* test or the Mann–Whitney *U* test, as appropriate, was used to compare the quantitative variables, and the Chi-square test or Fisher's exact test, as appropriate, was used to compare the qualitative variables between groups. Levels of significance: $p = 0.05$.
ALT, alanine aminotransferase; AST, aspartate aminotransferase; BLV, bulevirtide; FIB-4, Fibrosis-4 score; GGT, gamma-glutamyl transferase; INSTI, integrase inhibitor; NNRTI, non-nucleoside reverse transcriptase inhibitor; NUC, nucleos(t)ide analog; pegIFNα, pegylated interferon-α; qHBsAg, quantitative HBsAg; TAF/FTC, tenofovir disoproxil fumarate/emtricitabine; TDF/FTC, tenofovir disoproxil fumarate/emtricitabine.
*Number of missing data.

4–161 weeks). The characteristics of patients with early termination are indicated in Table S1.

Virological and biochemical results

From baseline to Week 48, HDV RNA gradually decreased in both groups (Fig. 1). Virological evolution between Day 0 and Week 48 in the 30 patients who received 48 weeks of treatment was as follows: 6.1 log_{10} IU/ml at Day 0, 4.52 log_{10} IU/ml at Week 12, 3.36 log_{10} IU/ml at Week 24, 3.65 log_{10} IU/ml at Week 36, and 3.23 log_{10} IU/ml at Week 48 in patients receiving BLV monotherapy and 6.0 log_{10} IU/ml at Day 0, 1.35 log_{10} IU/ml at Week 12, 0.37 log_{10} IU/ml at Week 24, 0.22 log_{10} IU/ml at Week 36, and 2.02 log_{10} IU/ml at Week 48 in patients receiving BLV + pegIFNα (per-protocol analysis). Individual data kinetics for each treatment group are indicated in Fig. 2A and B. At Week 48, HDV RNA was not available in four patients.

At Week 48, 10 of 19 patients (52.6%) in the 2 mg BLV group and five of seven patients (71.4%) in the 2 mg BLV + pegIFNα group had reached undetectable HDV RNA or a 2 log_{10} IU/ml or greater decline in HDV RNA (per-protocol analysis). Virological response at Weeks 12, 24, and 48 is indicated in Fig. 3A and Fig. S1A.

At Week 48, eight of 19 patients (31.6%) in the 2 mg BLV group and four of seven patients (57.1%) in the 2 mg BLV + pegIFNα group had undetectable HDV RNA (per-protocol analysis) (Fig. 3B and Fig. S1B).

From baseline to Week 48, ALT levels are shown in Fig. 3C and Fig. S1C. At Week 48, ALT level was normal or <40 IU/L in nine patients (42.9%) in the 2 mg BLV group and in four patients (66.7%) in the 2 mg BLV + pegIFNα group (per-protocol analysis) (Fig. 3C and Fig. S1C). No ALT flare or significant ALT elevation during treatment was observed.

At Week 48, seven of 19 patients (36.8%) in the 2 mg BLV group and three of six patients (50%) in the 2 mg BLV + pegIFNα group had a combined response (normal ALT level or <40 IU/L and undetectable HDV RNA or decline ≥2 log_{10} IU/ml from baseline) in per-protocol analysis (Fig. 3D and Fig. S1D).

Median FIB-4 score did not significantly change between Day 0 and Week 48, with values of 2.62 (IQR 1.5–3.88) and 2.10 (IQR 1.30–4.84), respectively ($p = 0.92$).

Follow up

After Week 48, 23 patients continued HDV treatment. Biochemical and virological responses are depicted in Fig. 4 (per-protocol analysis).

Table 2. Treatment discontinuation and main adverse events.

	BLV monotherapy (n = 27)	BLV + pegIFNα (n = 11)	*p* value
Discontinuation before Week 48, n (%)	4 (14.8)	4 (36.4)	0.1950
Duration of treatment before discontinuation (weeks), mean ± SD	31.1 ± 6.4	14.1 ± 8.5	0.0304
Causes of discontinuation before Week 48, n (%)			
Severe adverse events*	0 (0)	2 (50)	
Lost to follow-up	1 (25)	1 (25)	
Poor response or compliance	3 (75)	1 (25)	
All grade 3 and 4 adverse events*, n (%)	9 (42.9)	6 (100)	0.0200
Serious adverse events	9 (42.9)	4 (66.7)	0.3845
Increased bile acids (>15 N)	1 (4.8)	0	
Thrombocytopenia	1 (4.8)	2 (33.3)	
Neutropenia	1 (4.8)	5 (83.3)	
Gamma-glutamyl transferase increase	1 (4.8)	2 (33.3)	
Pruritus	3 (14.3)	1 (16.7)	

Qualitative variables are reported as n (%), and quantitative variables are reported as mean ± SD or median (IQR). The Mann–Whitney *U*-test was used to compare the quantitative variables, and the Chi-square test or Fisher's exact test, as appropriate, was used to compare the qualitative variables between groups. Levels of significance: *p* = 0.05.
BLV, bulevirtide; pegIFNα pegylated interferon-α.
*Number of patients who presented at least one event (available only in the ANRS HD EP01 BuleDelta cohort).

Among the 11 patients who received BLV + pegIFNα, seven continued BLV + pegIFNα after Week 48. Among them, five continued this association for more than 96 weeks, whereas the other two stopped pegIFNα at Weeks 71 and 81.

Among the 38 patients, 10 received 96 weeks of treatment. Among these 10 patients, eight had undetectable HDV RNA at week 96: five patients (62.5%) in the BLV group and three patients (37.5%) in the BLV + pegIFNα group.

Durability of virological response

Three patients with undetectable HDV RNA stopped treatment at Weeks 12, 24, and 48. Among them, a relapse was observed in two patients 12 weeks later. Only one patient, who received only 12 weeks of BLV + pegIFNα, had persistent undetectable HDV RNA at Week 36. At inclusion, HBV DNA was undetectable, and quantitative HBsAg (qHBsAg) was 437 IU/ml, respectively. At the end of treatment, HBV DNA was still undetectable. Unfortunately, information on qHBsAg was not available at the end of treatment or later.

Four patients stopped treatment after Week 48, with undetectable HDV RNA at end of treatment (three patients treated with BLV for 62, 64, and 67 weeks and one patient treated with BLV + pegIFNα for 71 weeks). None of them had sustained virological response.

HIV treatment and follow-up

Most patients (91.9%) received a three-drug combination for HIV treatment. Specifically, 8.1% of patients received lamivudine (3TC), 59.5% received tenofovir alafenamide/emtricitabine (TAF/FTC), 32.4% received tenofovir disoproxil fumarate/emtricitabine (TDF/FTC), 62.2% received an integrase inhibitor (INSTI), and 32.4% received a non-nucleoside reverse transcriptase inhibitor (NNRTI). The characteristics of patients with detectable HIV RNA are presented in Table S2. At treatment initiation, 10 patients (26.3%) had quantifiable HIV RNA (seven patients in the 2 mg BLV group and three in the BLV + pegIFNα group). At Week 48, four patients (19%) had detectable HIV RNA (two patients in each group).

Factors associated with virological response at week 48

By univariate analysis, no baseline factor was found to be significantly associated with virological response at Week 48 (age, sex, cirrhosis or not, HDV RNA viral load, treatment, ALT

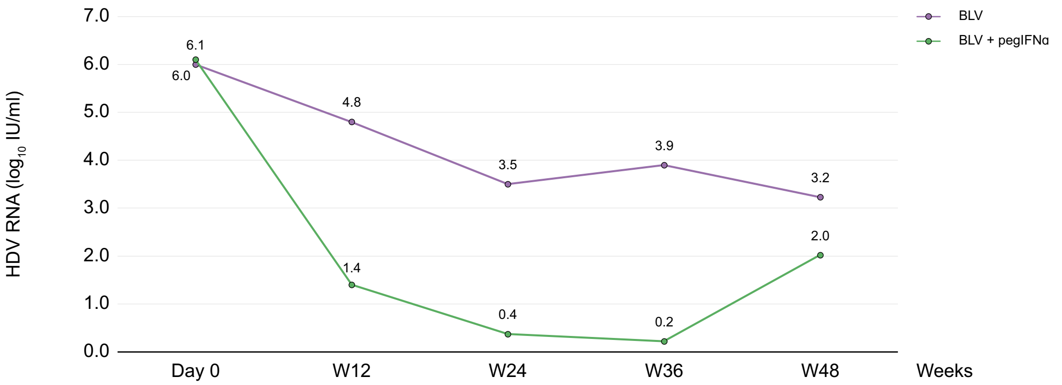

N° at risk					
BLV	19	18	18	18	19
BLV + pegIFNα	7	7	7	6	7
Missing	0	1	1	2	0

Fig. 1. Evolution of HDV RNA through Week 48. BLV, bulevirtide; pegIFNα, pegylated interferon-α.

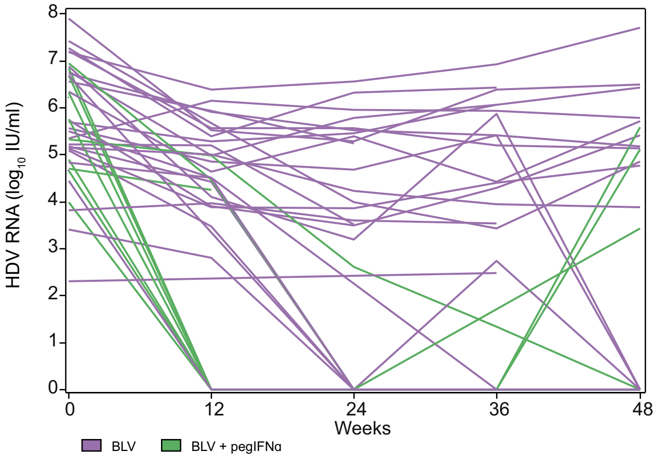

Fig. 2. Individual evolution of HDV RNA through Week 48. Patients treated with (A) BLV and (B) BLV + pegIFNα. BLV, bulevirtide; PEG-IFN, pegylated interferon-α.

level, and association with pegIFNα). However, normal ALT level at Week 12 was significantly associated with virological response at Week 48 (OR 8, 95% CI 1.21–52.69, p = 0.03).

Tolerance

Overall, 40 AEs were reported for 18 of 38 patients (47.4%). Only 13 patients had severe AEs (Table 2). Mean biliary acid levels were 14.55 + 18.55 µmol/L at Day 0, 50.33 + 39.82 µmol/L at Week 12, and 31.13 + 23.60 µmol/L at Week 48. Severe AEs were as follows: colorectal cancer, injection site reaction, pruritus, rash, biliary acid elevation, ALT or gamma-glutamyl transferase (GGT) increase, cardiovascular events, COVID-19 infection, lymphopenia, and neutropenia. No patient had liver decompensation.

Discussion

Currently, BLV 2 mg is the only EMA-approved drug for HDV treatment. To our knowledge, this study is the first to show that the treatment with BLV for 48 weeks in adults living with HIV with HDV infection, with or without pegIFNα, is well tolerated and safe, induces a significant virological response, and reduces ALT levels.

After 48 weeks of treatment, a virological response, defined as undetectable HDV RNA or HDV RNA decline >2 $\log_{10}$ from baseline, was observed in 15 of 26 patients (57.7%) in the per-protocol analysis. This result is consistent with findings from previous clinical trials with BLV treatment. Wedemeyer *et al.*[8] reported that 54% of patients achieved undetectable HDV RNA or an HDV RNA decline of >2 $\log_{10}$ from baseline with 2 mg BLV. In a recently published phase III study, the HDV RNA level at Week 48 was undetectable in 12% of patients in the 2 mg group.[9] In the present study, this rate was 38.5% in the per-protocol analysis.

The present study shows that combining BLV and anti-HIV drugs did not affect HIV viral load or CD4 count in this

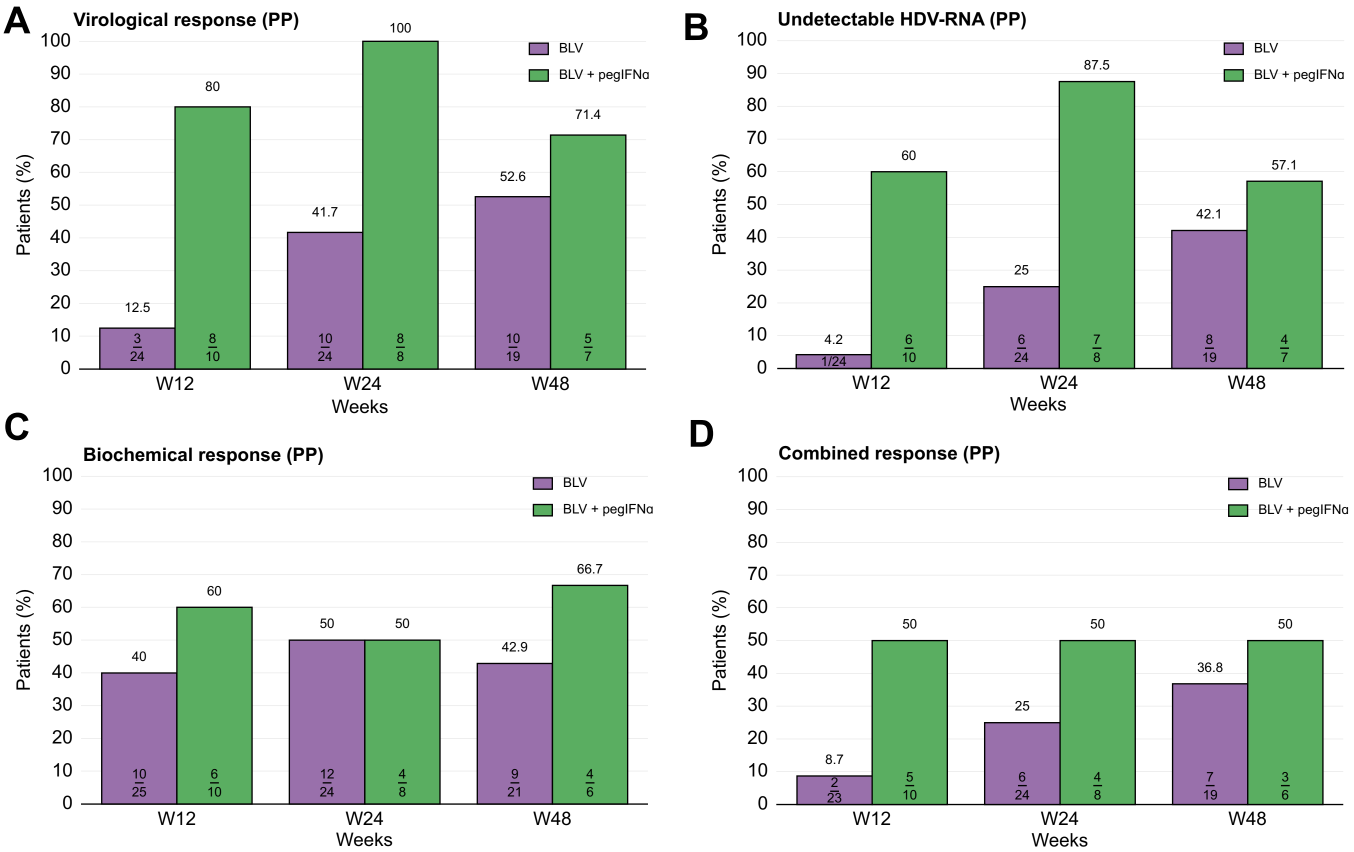

Fig. 3. Per-protocol analysis results at Weeks 12, 24, and 48. (A) Virological response (>2 $\log_{10}$ HDV RNA decline from baseline), (B) undetectable HDV RNA, (C) biochemical response (normal ALT level), and (D) combined response (normal ALT level and >2 $\log_{10}$ HDV RNA decline from baseline or undetectable HDV RNA). ALT, alanine aminotransferase; BLV, bulevirtide; pegIFNα, pegylated interferon-α; PP, per protocol.

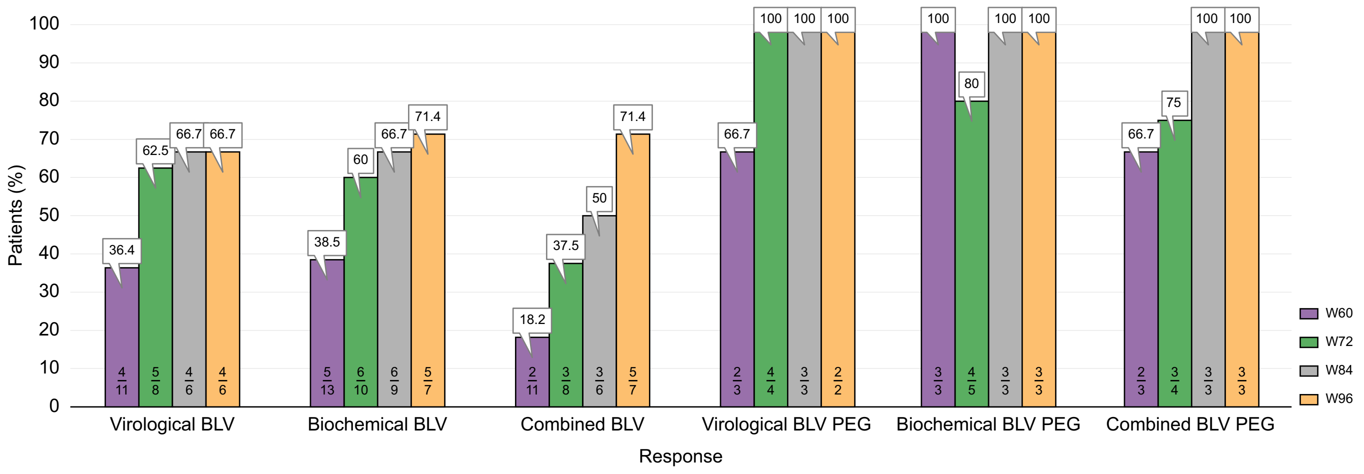

Fig. 4. Virological, biochemical, and combined response at Weeks 60, 72, 84, and 96 in patients who continued treatment after Week 48. BLV, bulevirtide; PEG-IFN, pegylated interferon-α; PP, per protocol.

population of adults living with HIV with HDV/HBV coinfection. We did not observe any worsening of HIV viral replication in these patients. Therefore, BLV could be used in HIV-infected patients without modification of three-drug combination regimens. Only one study evaluated BLV in five patients with HIV and HDV infection who received BLV for 11 months.[13] A combined response was achieved in 60% of those patients with HIV. In the present study with 38 patients, this was 40%.

In the MYR202 study, by Week 48 (24 weeks after BLV cessation), HDV RNA concentrations had rebounded, with median changes from Week 24 to Week 48 of 1.9 log10 IU/m.[8] Only 4% of patients had sustained virological response. This is similar to our observation that most patients relapsed after stopping BLV, with or without pegIFNα. In the present study, only one patient, who received only 12 weeks of BLV + pegIFNα, had a sustained virological response. This suggests that, currently, BLV therapy should not be stopped even in patients who achieved undetectable HDV RNA. More studies are needed to define response-guided therapy strategies. The recent EASL guidelines stated that the combination of pegIFNα and BLV may be considered in patients without pegIFNα intolerance or contraindications.[12]

In this study, we provide the first real-life results of efficacy and tolerance of a combined treatment (BLV + pegIFNα) in adults living with HIV. In our study population, a virological response at Week 48 was observed in 71% of patients receiving BLV + pegIFNα. In the BLV monotherapy group, most discontinuations of BLV treatment were related to the patient's social status (lost to follow-up or poor compliance). This result should be seen in the context of the patient population we see in France.[14] Most of these patients were in precarious situations, migrants, or homeless.

Between Weeks 36 and 48, we observed an increase in viral load in the group of patients receiving BLV + pegIFNα. This increase was not related to any reported change in pegIFNα dose and could be related to a lower compliance in this group because of the AEs of pegIFNα.

This study has limitations. Because this was a real-life study, no randomization was performed. The decision to combine BLV with pegIFNα was made by the physicians based on their own clinical judgment. Therefore, it cannot be excluded that pegIFNα was used in patients with less severe liver disease or in patients with no psychiatric history. Nevertheless, we did not observe any significant difference between the two groups of patients. We did not have the opportunity to evaluate HBsAg quantification in this real-life study. However, no substantial changes in HBsAg concentrations were observed in any of the BLV clinical trials.[9] One of the limitations of this study is related to its 'real-life' nature. For instance, relevant information on NUC therapy, albumin levels, or liver stiffness values were missing for a few patients.

In conclusion, this study showed that adults living with HIV coinfected with HBV/HDV can be treated by BLV with an overall good tolerability. This treatment can induce a virological response in more than 50% of patients, suggesting that BLV should be considered as a first-line therapy in this specific population. The combination of BLV and pegIFNα showed a strong virological response, suggesting that this combination could be used in patients without pegIFNα contraindication.

Affiliations

[1]*Hepatology Unit, Hôpital Haut Lévêque, Bordeaux University Hospital, Bordeaux, & INSERM U1312, Bordeaux University, Bordeaux, France;* [2]*CHU Rennes, Inserm, CIC 1414, Rennes, France;* [3]*Université Paris Cité; Centre Hospitalier Cochin Port Royal, DMU Cancérologie et spécialités médico-chirurgicales, Service d'Hépatologie, Paris, France;* [4]*Infectious Diseases Department, Hôpital de la Croix Rousse, Lyon University Hospitals, Lyon, France;* [5]*Sorbonne Université, Inserm IMPLESP, Infectious Diseases Unit, St Antoine Hospital, AP-HP, Paris, France;* [6]*Service d'Hépato-Gastroentérologie, Centre Hospitalier Universitaire, Grenoble, France;* [7]*Assistance Publique des Hôpitaux de Paris, Hôpital Saint-Louis, Service des Maladies Infectieuses et Tropicales, Paris, France;* [8]*Service d'Hépato-Gastroentérologie, Centre Hospitalier Universitaire, Dijon, France;* [9]*Service de Maladies Infectieuses, Centre Hospitalier Universitaire, Nantes, France;* [10]*Service d'Hépato-Gastroentérologie, Centre Hospitalier Inter-communal, Créteil, France;* [11]*Assistance Publique des Hôpitaux de Paris, Hôpital Bichat Claude Bernard, Service des Maladies Infectieuses et Tropicales, Paris, France;* [12]*Sorbonne Université, Institute of Cardiometabolism and Nutrition, Hospital Pitié Salpêtrière, Paris, France;* [13]*Hepatologie, Hôpital Avicenne, AP-HP, Avicenne, France;* [14]*Service d'Hépato-Gastroentérologie, Centre Hospitalier Universitaire, Montpellier, France;* [15]*Service d'Héatologie, AP-HP Henri Mondor, Créteil, France;* [16]*Hepato-gastroenterology Department, University Hospital Center and INSERM U 1248, Limoges University, Limoges, France;* [17]*Service d'Hépato-Gastroentérologie, Centre Hospitalier Universitaire, Lille, France;* [18]*France Assistance Publique des Hôpitaux de Paris, Hôpital Tenon, Service*

des Maladies Infectieuses et Tropicales, Paris, France; [19]CHU Rennes, Service des Maladies du Foie, Rennes, France; [20]Service d'Hépato-Gastroentérologie, Centre Hospitalier Universitaire, Toulouse, France; [21]Hospices Civils de Lyon, Hôpital Edouard Herriot, Fédération des Spécialités digestives, et Université Claude Bernard Lyon 1, Lyon, France; [22]Service d'Hépato-Gastroentérologie, Centre Hospitalier Général, Avignon, France; [23]Université Paris-Cité, Centre de recherche sur l'inflammation, Inserm U1149, Department of Hepatology, Assistance Publique des Hôpitaux de Paris (AP-HP), Hôpital Beaujon, Clichy, France; [24]France Assistance Publique des Hôpitaux de Paris, Hôpital Paul Brousse, Service d'Hépatologie, Villejuif, France; [25]Service d'Hépato-Gastroentérologie, Centre Hospitalier Général, Saint-Brieuc, France; [26]Sorbonne University, Infectious Diseases Department, Pitié-Salpêtrière Hospital, AP-HP, Pierre Louis Epidemiology and Public Health Institute (iPLESP), INSERM U1136, Paris, France; [27]Service de Virologie, Hôpital de la Croix Rousse, Hospices Civils de Lyon, Lyon, France; [28]National Reference Centre for Viral Hepatitis B, C and Delta, Department of Virology, Paris-Seine-Saint-Denis University Hospitals, Bobigny, France; [29]ANRS MIE, PariSanté Campus, 2 rue d'Oradour sur Glane, Paris, France; [30]Hepatology Department, Hospices Civils de Lyon, INSERM U1052-CRCL; Université Claude Bernard Lyon 1, Lyon, France

Abbreviations

AE, adverse event; ALT, alanine aminotransferase; AST, aspartate aminotransferase; BLV, bulevirtide; GGT, gamma-glutamyl transferase; INSTI, integrase inhibitor; NNRTI, non-nucleoside reverse transcriptase inhibitor; NUC, nucleos(t)ide analog; OR, odds ratio; pegIFNα, pegylated interferon-α; qHBsAg, quantitative HBsAg; SAE, serious adverse event; TAF/FTC, tenofovir disoproxil fumarate/emtricitabine; TDF/FTC, tenofovir disoproxil fumarate/emtricitabine.

Financial support

The authors did not receive any financial support to produce this manuscript.

Conflicts of interest

VdL: AbbVie, Gilead, Orphalan, GSK, BMS, Novo Nordisk, Bayer, Janssen, eScopics, Alfasigma, and AstraZeneca. EB: Gilead and Merck. KL: MSD, ViiV Healthcare, Gilead, Abbvie, Roche, Moderna, Chiesi and GSK. VL-R: Abbvie, Gilead and Ipsen. DA: ViiV, MSD and Gilead. TA: Antios Therapeutics, AbbVie, Eiger Bio-Pharmaceuticals, Enyo Pharma, Gilead Sciences, GSK, Janssen, and Roche. DR: Gilead. All other authors have no conflicts of interest to declare.

Please refer to the accompanying ICMJE disclosure forms for further details.

Authors' contributions

Conception and design of the study, inclusion of patients, and writing the manuscript draft: VdL. Conceptualization, methodology, software supervision, project administration, and data collection and analysis: CF-L, ELP, CT, AK. Funding: FC, ER. Inclusion of patients and review of the final manuscript: SP, DA, KL, M-NH, CL-C, AM, EB, IR, AG, VR, NG, G-PP, VL, VL-R, PM, JC, CJ, SM, JD, J-PA, TA, BR, ALG, M-AV, CS, EG, CT, AK, FC, ER, MS-L, DR, FZ. Reviewed the final version and approved for submission: all authors.

Data availability statement

The ANRS HD EP01 BuleDelta cohort is a French nationwide cohort sponsored by the ANRS (France REcherche Nord & sud Sida-hiv Hepatites). Data are owned by ANRS, and there are also legal restrictions to share data publicly. Nonetheless, data can be accessed upon demand to the scientific committee and the ANRS, which can allow a contractual assessment for collaboration purposes. Applicants will be asked to complete a Research Application Form specifying details for their planned study, which will then be reviewed by the ANRS HD EP01 BuleDelta scientific committee. The ANRS HD EP01 BuleDelta cohort is eager to promote collaboration among researchers and to see our unique database and biobank of patients coinfected with HDV–HBV used in studies that meet our ethics and consenting process.

ANRS HD EP01 BuleDelta scientific committee

Marc Bourlière, Emmanuel Gordien, Fabienne Marcellin, Fatoumata Coulibaly, Victor de Lédinghen, Catherine Gaudy-Graffin, Jeremie Guedj, Michelle Sizorn, Marianne L'Hénaff, Karine Lacombe, Elise Landry, Estelle Le Pabic, Massimo Levrero, Manal Mecheri, Ventzislava Petrov-Sanchez, Alain Renault, Dominique Roulot, Caroline Scholtès, Lawrence Serfaty, Miroslava Subic-Levrero, Christelle Tual, Fabien Zoulim.

ANRS HD EP01 BuleDelta study group

François Raffi, Laurent Alric, Patrick Miailhes, Albert Tran, Christiane Stern, Xavier Causse, Simona Tripon, Ghassan Riachi, Olivier Chazouillères, Armando Abergel, Louis d'Alteroche, Jérôme Gournay, Garance Lagadic, Patrizia Carrieri, Ségolène Brichler, Martin Siguier, Jessica Krause, Juliette Foucher, Souad Ben Ali, Magdalena Meszaros, Anne Varaut, Valérie Canva.

Supplementary data

Supplementary data to this article can be found online at https://doi.org/10.1016/j.jhepr.2024.101057.

References

Author names in bold designate shared co-first authorship

[1] Asselah T, Rizzetto M. Hepatitis D virus infection. N Engl J Med 2023;389:58–70.
[2] Piroth L, Pol S, Lacombe K, et al. Management and treatment of chronic hepatitis B virus infection in HIV positive and negative patients: the EPIB 2008 study. J Hepatol 2010;53:1006–1012.
[3] Stockdale AJ, Kreuels B, Henrion MYR, et al. The global prevalence of hepatitis D virus infection: systematic review and meta-analysis. J Hepatol 2020;73:523–532.
[4] Alfaiate D, Clément S, Gomes D, et al. Chronic hepatitis D and hepatocellular carcinoma: a systematic review and meta-analysis of observational studies. J Hepatol 2020;73:533–539.
[5] **Wedemeyer H**, **Yurdaydin C**, Hardtke S, et al. Peginterferon alfa-2a plus tenofovir disoproxil fumarate for hepatitis D (HIDIT-II): a randomised, placebo controlled, phase 2 trial. Lancet Infect Dis 2019;19:275–286.
[6] Martin P, Jacobson IM. New therapies and management options for hepatitis D. Am J Gastroenterol 2023;118:1105–1107.
[7] Lampertico P, Roulot D, Wedemeyer H. Bulevirtide with or without pegIFNα for patients with compensated chronic hepatitis delta: from clinical trials to real-world studies. J Hepatol 2022;77:1422–1430.
[8] Wedemeyer H, Schöneweis K, Bogomolov P, et al. Safety and efficacy of bulevirtide in combination with tenofovir disoproxil fumarate in patients with hepatitis B virus and hepatitis D virus coinfection (MYR202): a multicentre, randomised, parallel-group, open-label, phase 2 trial. Lancet Infect Dis 2023;23:117–129.
[9] Wedemeyer H, Aleman S, Brunetto MR, et al. A phase 3, randomized trial of bulevirtide in chronic hepatitis D. N Engl J Med 2023;389:22–32.
[10] Dietz-Fricke C, Tacke F, Zöllner C, et al. Treating hepatitis D with bulevirtide – real-world experience from 114 patients. JHEP Rep 2023;5:100686.
[11] Degasperi E, Anolli MP, Uceda Renteria SC, et al. Bulevirtide monotherapy for 48 weeks in patients with HDV-related compensated cirrhosis and clinically significant portal hypertension. J Hepatol 2022;77:1525–1531.
[12] European Association for the Study of the Liver. EASL Clinical Practice Guidelines on hepatitis delta virus. J Hepatol 2023;79:433–460.
[13] Visco Comandini U, De Santis E, De Maria F, et al. "Real world" efficacy of bulevirtide in HBV/HDV-related cirrhosis including people living with HIV: results from the compassionate use programme at INMI Spallanzani in Rome, Italy. HIV Med 2023;24:1075–1082.
[14] **Roulot D**, **Brichler S**, Layese R, et al. Origin, HDV genotype and persistent viremia determine outcome and treatment response in patients with chronic hepatitis delta. J Hepatol 2020;73:1046–1062.

Keywords: Cirrhosis; HDV; HBV; Pegylated interferon; Hepatitis D; HDV RNA; HIV; HIV RNA; Entry inhibitors; HBV DNA.
Received 18 October 2023; Received in revised form 22 February 2024; Accepted 27 February 2024; Available online 26 March 2024

Journal of Hepatology, Volume *6*

Supplemental information

Treatment with bulevirtide in HIV-infected patients with chronic hepatitis D: ANRS HD EP01 BuleDelta and compassionate cohort

Victor de Lédinghen, Claire Fougerou-Leurent, Estelle Le Pabic, Stanislas Pol, Dulce Alfaiate, Karine Lacombe, Marie-Noëlle Hilleret, Caroline Lascoux-Combe, Anne Minello, Eric Billaud, Isabelle Rosa, Anne Gervais, Vlad Ratziu, Nathalie Ganne, Georges-Philippe Pageaux, Vincent Leroy, Véronique Loustaud-Ratti, Philippe Mathurin, Julie Chas, Caroline Jezequel, Sophie Métivier, Jérôme Dumortier, Jean-Pierre Arpurt, Tarik Asselah, Bruno Roche, Antonia Le Gruyer, Marc-Antoine Valantin, Caroline Scholtès, Emmanuel Gordien, Christelle Tual, Amel Kortebi, Fatoumata Coulibaly, Eric Rosenthal, Miroslava Subic-Levrero, Dominique Roulot, Fabien Zoulim, and the ANRS HD EP01 BuleDelta study group

Treatment with bulevirtide in HIV-infected patients with chronic hepatitis D: ANRS HD EP01 BuleDelta and compassionate cohort

Victor de Lédinghen, Claire Fougerou-Leurent, Estelle Le Pabic, Stanislas Pol, Dulce Alfaiate, Karine Lacombe, Marie-Noëlle Hilleret, Caroline Lascoux-Combe, Anne Minello, Eric Billaud, Isabelle Rosa, Anne Gervais, Vlad Ratziu, Nathalie Ganne, Georges-Philippe Pageaux, Vincent Leroy, Véronique Loustaud-Ratti, Philippe Mathurin, Julie Chas, Caroline Jezequel, Sophie Métivier, Jérôme Dumortier, Jean-Pierre Arpurt, Tarik Asselah, Bruno Roche, Antonia Le Gruyer, Marc-Antoine Valantin, Caroline Scholtès, Emmanuel Gordien, Christelle Tual, Amel Kortebi, Fatoumata Coulibaly, Eric Rosenthal, Miroslava Subic-Levrero, Dominique Roulot, Fabien Zoulim, and the ANRS HD EP01 BuleDelta study group

Table of contents

Fig. S1……………………………………………………………………………2

Table S1…………………………………………………………………………5

Table S2…………………………………………………………………………6

Table S3…………………………………………………………………………7

Supplementary references……………………………………………………..8

Fig. S1. Virological and/or biochemical response (intent to treat analysis)

A Virological response ($\geq$ 2 log_{10} HDV RNA decline from baseline) at week 12, 24 and 48

B Undetectable HDV RNA at week 12, 24 and 48

C Biochemical response (normal ALT level) at week 12, 24 and 48

D Combined response (normal ALT level and $\geq$ 2 log_{10} HDV RNA decline from baseline or undetectable HDV RNA) at week 12, 24 and 48

BLV. Bulevirtide

PEG-IFN. Pegylated interferonα

Supplementary file 1A

Virological response (ITT)

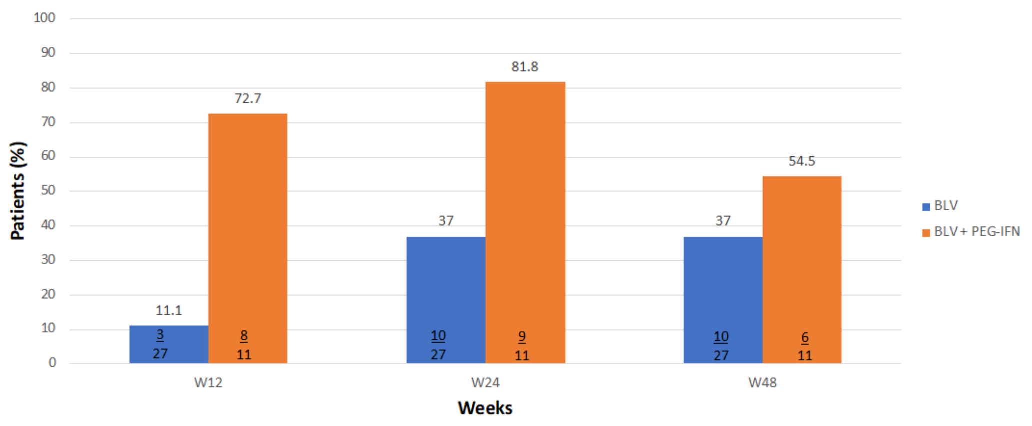

Supplementary file 1B

Undetectable HDV-RNA (ITT)

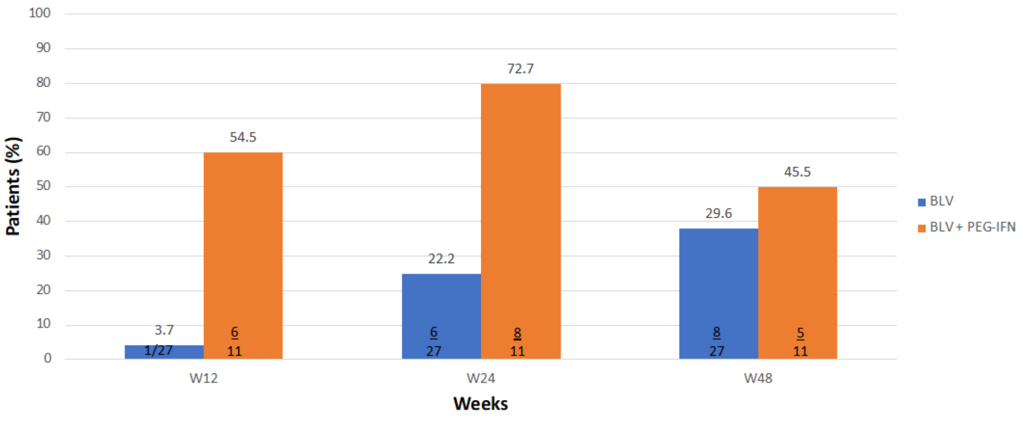

Supplementary file 1C

Biochemical response (ITT)

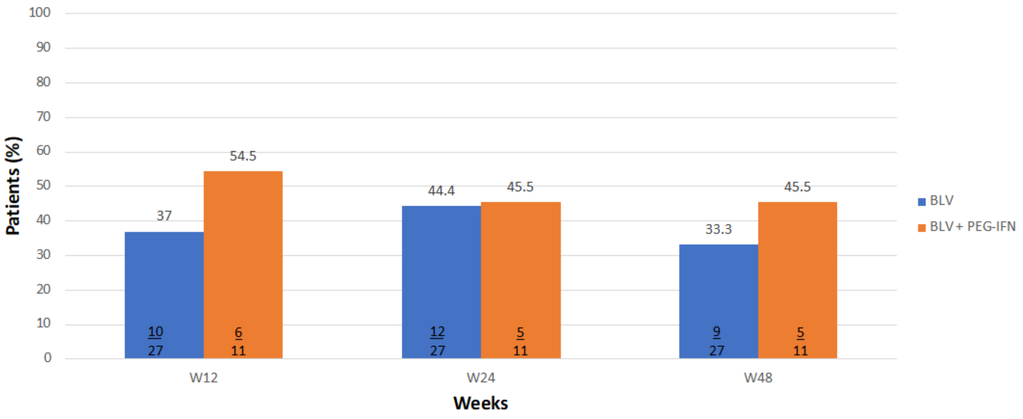

Supplementary file 1D

Combined response (ITT)

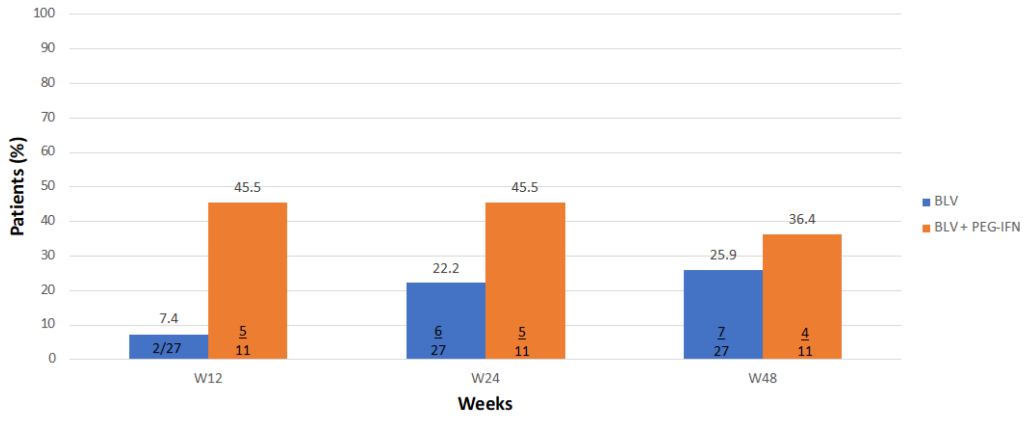

Table S1. Characteristics of Patients discontinuing antiviral therapy.

ID	Gender	Age (years)	HDV treatment	LSM (kPa)	Cirrhosis	HDV RNA at D0 (log_{10} IU/ml)	ALT level at D0 (IU/L)	Treatment duration (weeks)	HDV RNA at the end of treatment (log_{10} IU/ml)	ALT level at the end of treatment (IU/L)	Cause of discontinuation	HDV-RNA at FU12 (log_{10} IU/ml)	ALT at FU12 (IU/L)
1	Female	56	BLV	NA	Yes	7.27	57	35.86	6.44	20.5	Virological failure	6.96	78.9
2	Male	54	BLV + pegIFN α	75	Yes	6.22	100	3.86	6.33	65	Variceal bleeding	NA	NA
3	Male	52	BLV + pegIFN α	NA	No	4.71	63	16.86	4.26	46	NA	3.68	68
4	Female	55	BLV	20.9	Yes	5.22	62	25.86	3.51	33	Patient wish	NA	NA
5	Male	55	BLV	22	Yes	6.57	81	25.43	5.24	55	Lost to FU	NA	NA
6	Male	35	BLV	5.7	No	5.70	145	37.43	6.06	66	No compliance	5.69	95
7	Male	51	BLV + pegIFN α	70.1	Yes	4.67	36	24.00	0	21	Adverse events	4.36	70
8	Male	42	BLV + pegIFN α	22	Yes	3.99	75	11.71	0	42	Prison	0	33

FU: follow-up
LSM: liver stiffness measurement

Table S2. Characteristics of 11 patients with detectable HIV RNA at initiation of treatment or W48.

ID	Gender	Age (years)	HDV treatment	HIV RNA at D0 (cp/ml)	HIV RNA level detection (cp/ml)	CD4 at D0 (/mm^3)	HIV RNA at W48 (cp/ml)	CD4 at W48 (/mm^3)	HIV treatment
1	Male	55	BLV	0	20	480	30	524	TAF/FTC + INSTI + NNRTI
2	Male	30	BLV	65	20	656	0	735	TAF/FTC + INSTI
3	Male	59	BLV	33	20	128	NA	NA	TDF/FTC + INSTI
4	Male	39	BLV + pegIFNα	30	20	843	70	1033	TAF/FTC + NNRTI
5	Male	52	BLV + pegIFNα	21	20	710	NA	NA	TDF/FTC + protease inhibitor
6	Female	56	BLV	30	20	NA	NA	NA	TAF/FTC + INSTI
7	Male	55	BLV	50	20	375	0	293	TAF/FTC + INSTI
8	Female	48	BLV	123	20	588	238	544	TDF/FTC + INSTI + CCR5 antagonist
9	Female	41	BLV	20	20	317	NA	NA	TDF/FTC + protease inhibitor
10	Male	45	BLV	19000	20	179	NA	NA	3TC + INSTI
11	Male	51	BLV + pegIFNα	31	20	500	54	470	TAF/FTC + INSTI

INSTI: Integrase strand transfer inhibitor
NNRTI : Non-nucleoside reverse ratzscriptase inhibitor
BLV: Bulevirtide; BLV + pegIFNα: Bulevirtide + pegylated interferon alfa;
TAF: Tenofovir alafenamide; FTC: Emtricitabine; TDF: Tenofovir Disoproxil Fumarate; CCR5: Chemokine receptor 5; 3TC: Lamivudine; NA: Not available

Table S3. Characteristics of the 11 patients with no response or partial response at week 48.

ID	Gender	Age (years)	HDV treatment	LSM (kPa)	Cirrhosis	HDV RNA at D0 (log10 IU/ml)	HDV RNA at W48 (log10 IU/ml)	HDV RNA at W60 (log10 IU/ml)	HDV RNA at W72 (log10 IU/ml)	HDV RNA at EOT (log10 IU/ml)
		Non responder patients								
1	Male	55	BLV	38.5	Yes	5.74	5.42	6.07	NA	
2	Male	44	BLV	NA	Yes	5.36	5.78	NA	NA	
3	Male	40	BLV	NA	Yes	6.78	6.44	5.52	NA	
4	Female	35	BLV	7.9	No	7.19	7.71	5.16	0	
5	Male	33	BLV	NA	No	6.35	5.71	NA	NA	6.52 (W87)
6	Female	48	BLV	9.9	Yes	5.15	4.48	5.29	NA	
		Partial responder patients								
7	Male	39	BLV+ pegIFNα	4.2	No	6.85	5.60	NA	4.60	
8	Male	58	BLV	6.1	Yes	6.74	5.19	5.75	6.05	
9	Male	55	BLV	48.8	Yes	7.90	6.5	5.95	NA	
10	Female	42	BLV+ pegIFNα	9.5	No	6.32	5.12	NA	NA	5.30 (W59)
11	Male	46	BLV	15	Yes	5.17	3.89	4.32	4.10	

*EOT = End Of Treatment

LSM: liver stiffness measurement

Supplementary references

1. Asselah T, Rizzetto M. Hepatitis D Virus Infection. N Engl J Med 2023;389:58-70.
2. Piroth L, Pol S, Lacombe K, et al. Management and treatment of chronic hepatitis B virus infection in HIV positive and negative patients: the EPIB 2008 study. J Hepatol 2010;53:1006-1012.
3. Stockdale AJ, Kreuels B, Henrion MYR, et al. The global prevalence of hepatitis D virus infection: Systematic review and meta-analysis. J Hepatol 2020;73:523-532.
4. Alfaiate D, Clement S, Gomes D, et al. Chronic hepatitis D and hepatocellular carcinoma: A systematic review and meta-analysis of observational studies. J Hepatol 2020;73:533-539.
5. **Wedemeyer H, Yurdaydin C**, Hardtke S, et al. Peginterferon alfa-2a plus tenofovir disoproxil fumarate for hepatitis D (HIDIT-II): a randomised, placebo controlled, phase 2 trial. Lancet Infect Dis 2019;19:275-286.
6. Martin P, Jacobson IM. New Therapies and Management Options for Hepatitis D. Am J Gastroenterol 2023;118:1105-1107.
7. Lampertico P, Roulot D, Wedemeyer H. Bulevirtide with or without pegIFNalpha for patients with compensated chronic hepatitis delta: From clinical trials to real-world studies. J Hepatol 2022;77:1422-1430.
8. Wedemeyer H, Schoneweis K, Bogomolov P, et al. Safety and efficacy of bulevirtide in combination with tenofovir disoproxil fumarate in patients with hepatitis B virus and hepatitis D virus coinfection (MYR202): a multicentre, randomised, parallel-group, open-label, phase 2 trial. Lancet Infect Dis 2023;23:117-129.
9. Wedemeyer H, Aleman S, Brunetto MR, et al. A Phase 3, Randomized Trial of Bulevirtide in Chronic Hepatitis D. N Engl J Med 2023;389:22-32.
10. Dietz-Fricke C, Tacke F, Zollner C, et al. Treating hepatitis D with bulevirtide - Real-world experience from 114 patients. JHEP Rep 2023;5:100686.
11. Degasperi E, Anolli MP, Uceda Renteria SC, et al. Bulevirtide monotherapy for 48 weeks in patients with HDV-related compensated cirrhosis and clinically significant portal hypertension. J Hepatol 2022;77:1525-1531.
12. European Association for the Study of the Liver. Electronic address eee, European Association for the Study of the L. EASL Clinical Practice Guidelines on hepatitis delta virus. J Hepatol 2023;79:433-460.
13. Visco Comandini U, De Santis E, De Maria F, et al. "Real world" efficacy of bulevirtide in HBV/HDV-related cirrhosis including people living with HIV: Results from the compassionate use programme at INMI Spallanzani in Rome, Italy. HIV Med 2023; Oct;24(10):1075-1082.
14. **Roulot D, Brichler S**, Layese R, et al. Origin, HDV genotype and persistent viremia determine outcome and treatment response in patients with chronic hepatitis delta. J Hepatol 2020;73:1046-1062.
